# C5b-9-Targeted Molecular MR Imaging in Rats with Heymann Nephritis: A New Approach in the Evaluation of Nephrotic Syndrome

**DOI:** 10.1371/journal.pone.0121244

**Published:** 2015-03-16

**Authors:** Qiang Huang, Song Wen, Bo Wang, Qidong Wang, Chuangen Guo, Xinying Wu, Rui Zhang, Rong Yang, Feng Chen, Wenbo Xiao

**Affiliations:** 1 Department of Radiology, The First Affiliated Hospital, School of Medicine, Zhejiang University, Hangzhou, China; 2 Jiangsu Key Laboratory of Molecular and Functional Imaging, Medical School, Southeast University, Nanjing, China; 3 Department of Pathology, The First Affiliated Hospital, School of Medicine, Zhejiang University, Hangzhou, China; 4 Department of Radiology, Nanjing First Hospital, Nanjing Medical University, Nanjing, China; National Centre for Scientific Research “Demokritos”, GREECE

## Abstract

Membranous nephropathy (MN) is the major cause of adult nephrotic syndrome, which severely affects patients’ quality of life. Currently, percutaneous renal biopsy is required to definitively diagnose MN. However, this technique is invasive and may cause severe complications. Therefore, an urgent clinical need exists for dynamic noninvasive monitoring of the renal state. In-depth molecular imaging studies could assist in finding a solution. Membrane attack complex C5b-9 is the key factor in the development of MN, and this protein primarily deposits in the glomerulus. The present study bound polyclonal antibodies to C5b-9 with ultrasmall superparamagnetic iron oxide (USPIO) nanoparticles to obtain C5b-9-targeted magnetic resonance molecular imaging probes. The probes were injected intravenously into rats with Heymann nephritis, a classic disease model of MN. The signal intensity in the T2*-weighted imaging of kidneys *in vivo* using 7.0 Tesla magnetic resonance imaging decreased significantly 24 hours after injection compared to the untargeted and control groups. This signal change was consistent with the finding of nanoparticle deposits in pathological glomeruli. This study demonstrated a novel molecular imaging technique for the assessment of MN.

## Introduction

Membranous nephropathy (MN) is the major cause of adult nephrotic syndrome, and the pathogenesis of MN has not been yet fully elucidated. Auto-antibodies attack the membrane antigens of glomerular epithelial cells (GECs) and induce glomerular injuries in MN. Immune complexes are dropped from GECs to the glomerular basement membrane (GBM), and primary immune complexes form under the epidermis. The immune complex sediments induce complements to produce C5b-9, which then activates a signaling pathway that causes GEC injuries and GBM damage, leading to albuminuria. Most studies have shown that C5b-9 is the key factor for MN development, which plays a decisive role in the formation of albuminuria [1,2,3]. Heymann nephritis (HN) exhibits a pathogenesis similar to MN, and HN is a well-accepted model for the study of MN in humans [4,5].

Percutaneous renal biopsy is required in clinical practice to definitively diagnose MN [6]. However, some patients with MN do not accept this invasive procedure due to its complication risks, including bleeding, infection, massive hemorrhage and septicemia. Moreover, biopsy fails to monitor the disease activity and therapeutic effects [7,8]. The kidney is an organ with an abundant blood supply, and it has strong compensation abilities. The kidney might have already been in an irreversible stage of fibrosis when the abnormal clinical features or positive laboratory findings emerge. Therefore, an urgent clinical need exists to develop a simple and noninvasive method that can be utilized to diagnose the disease and monitor its progression. The development of molecular magnetic resonance imaging (MRI) provides new opportunities to monitor pathological changes in kidneys *in vivo*. MRI contrast agents, such as ultrasmall superparamagnetic iron oxide (USPIO), can be combined with specific antibodies to form complex-targeting molecular probes. The probes are introduced into the body, and the antibodies specifically bind to the complex. Therefore, the magnetization properties of USPIO in the probes may change the T1/T2 relaxation time of tissues, and specific MRI findings of the targeting complex can be obtained [9]. The C5b-9 complex is a key factor in the development of MN and is primarily deposited in the glomerulus. Therefore, we presumed that molecular MRI that targets C5b-9 could be applied to evaluate the conditions of kidneys with MN.

This study bound an antibody to C5b-9 using USPIO nanoparticles to generate a C5b-9-targeting MRI probe (anti-C5b-9-USPIO). We injected the probe intravenously into rats with passive HN with the objective of studying whether the C5b-9-targeting probe could feasibly evaluate the pathological progress in the kidneys of rats using *in vivo* MRI at an ultrahigh field strength in a 7.0 Tesla MRI scanner.

## Materials and Methods

### Nanoparticle preparation and properties

USPIOs were provided by Beijing Oneder Hightech. Co. Beijing, China. A rabbit anti-human anti-C5b-9 polyclonal antibody and a nonspecific mouse IgG antibody were purchased commercially (Biosynthesis Biotechnology Co., Beijing, China). The synthetic process of the targeting probe is described briefly as follows. One milligram of PEG-coated USPIO was dissolved in boric acid buffer (pH = 9, 500 μl). One milligram of 1-ethyl-3-(3-dimethylaminopropyl) carbodiimide (EDC) and 0.5 mg of N-hydroxysuccinimide (NHS) were added and stirred at room temperature for 30 min. Finally, 200 μg of anti-C5b-9 was added and agitated slightly at room temperature for 3 h. After reaction, the liquid was diluted in PBS (pH 7.4) and purified with three passes in a centrifugal filter device. The purified probe was again suspended in PBS at a concentration of 1 mg/ml. The synthetic processing method of untargeted IgG-USPIO was identical to the above description.

TEM (JEOL-100CX) was used to detect the appearance of magnetic nanoparticles. Dynamic light scattering (DLS, 90 Plus Particle Size Analyzer; Brookhaven Instruments) was adopted to detect the magnetic nanoparticle hydrodynamic size and the stability of the probe. A vibrating sample magnetometer (Lakeshore 7407) was used to investigate the magnetic properties of the iron oxide nanoparticles. The T2 and T1 relaxation times of the nanoparticles were detected using an operating frequency of 128 MHz in a clinical 3.0 Tesla MRI (Achieva, Philips, Netherlands).

### Animal models

All animal experimental protocols were reviewed and approved by the experimental animal ethics committee of the school of medicine, Zhejiang University, Hangzhou, China, and were performed in accordance with the National Institutes of Health guidelines on animal care. All rats were housed two per cage in a temperature-controlled room (22–25°C) on a 12-h light/dark cycle with free access to food and water before and after tail injection. The health condition was monitored every four hours after USPIO injection. All surgeries were performed under general anesthesia (xylazine, 4 mg/kg; ketamine 75 mg/kg IM.) and euthanized with pentobarbital sodium (150 mg/kg IP.). All efforts were made to minimize the animals suffering. Rats with passive HN were prepared according to the methods of Malathi et al. [10] and Lotan et al. [11]. Briefly, brush border membranes (BBM) of mouse proximal tubules were extracted accordingly [10], and rabbit anti-mice BBM antiserum was prepared accordingly [11].

### Study design

**Fig 1:**
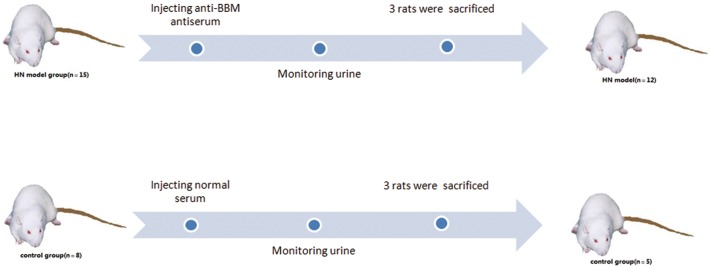
Brief procedure of Heymann nephritis models establishing. Twenty-three healthy male SD rats were randomly divided into two groups: 15 rats in the HN model group and 8 rats in the control group. The HN model rats were injected with rabbit anti-BBM antiserum. The control rats were injected with normal rabbit blood serum. The urine of each rat was monitored. At the end of the eighth week, 3 rats were randomly chosen from each group and sacrificed for renal pathology.

Twenty-three healthy male Sprague-Dawley rats weighing 170±20 g were randomly divided into two groups: 15 rats in the HN model group and 8 rats in the control group. All of the rats in the model group were injected simultaneously with rabbit anti-BBM antiserum at a dose of 0.6 ml/100 g through the tail veins. The control rats were injected with normal rabbit blood serum in the same manner. All of the rats were moved to metabolic cages at the end of the first, fourth and eighth week after initial immunity. The animals were subjected to a fasting state with free access to water. The urine of each rat was collected for 24 hours, and the total urinary protein content was measured. At the end of the eighth week, 3 rats were randomly chosen from each group and sacrificed for pathological examination of the kidneys. Therefore, 12 rats with HN and 5 normal SD rats remained and were used for *in vivo* MRI scanning. The brief procedure of animal models establishing was illustrated in Fig. 1.

Nine weeks after the onset of the HN model and verification using pathology, the remaining 12 HN rats were randomly divided into targeting and untargeted groups (n = 6 in each group). Five normal SD rats remained in the control group. Anti-C5b-9-USPIO was intravenously administered via the tail veins at a dose of 15 mg/kg in the targeting and control groups, and nonspecific IgG-USPIO was intravenously administered at the same dose to the untargeted group.

### *In vivo* MRI scanning

**Fig 2:**
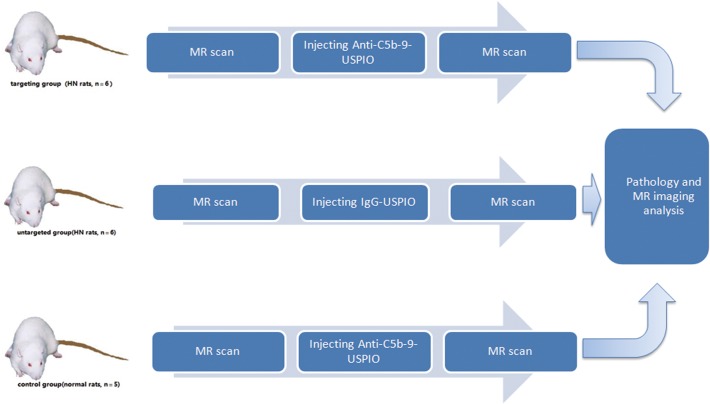
Three animal groups and brief experimental steps. Tweleve HN rats were randomly divided into targeting and untargeted groups (n = 6 in each group). Five normal SD rats were used as the control group. Anti-C5b-9-USPIO was intravenously injected into the targeting and control groups, and nonspecific IgG-USPIO was injected into the untargeted group. MRI sessions were performed before injection and 24 hours after injection.

All *in vivo* MRI scans were performed in a 7.0 Tesla micro-MRI system (Bruker, Rheinstetten, Germany) using a 35-mm birdcage coil with an interior diameter of 16 cm at horizontal scanning. The animals were anesthetized with a 4% isoflurane/air mixture that was delivered through a nose cone; they were then maintained under anesthesia with a 1.5–2% isoflurane/air mixture. The heart and respiratory rates of the rats were monitored. Thereafter, MRI scans of both kidneys were acquired using a sequence of 2-dimensional, fast low angle shots (FLASH) and the following parameters: repetition time (TR) ms/echo time (TE) ms, 250/5; Flip angle, 20°; section thickness, 0.5 mm; no intersection distance; number of sections, 20; FOV, 60 mm×60 mm; matrix, 256×256; minimum resolution, 98 μm×98 μm; total acquisition time, 15 min. MRI sessions were performed before injection and 24 hours after injection. The animal groups management and MR scan steps were shown in Fig. 2.

### Image analysis

**Fig 3:**
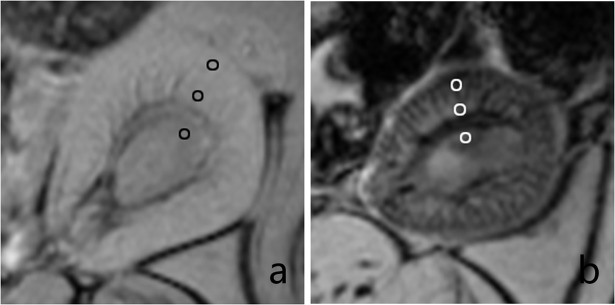
Samples of the ROIs. T2*WI pictures of a kidney in targeting group are shown. a, before injection. b, after injection. Significant signal decrease of the cortex was observed in Fig. 3B. The cortex, outer medulla, and inner medulla were measured in proper order from the outer compartment to inner compartment.

Two experienced MRI diagnosticians analyzed the images using dedicated software (ParaVision 5.0, Bruker, Germany). Signals of the kidney cortex, outer medulla, inner medulla and neighboring paravertebral muscles were measured in the same region of interest. The ROIs were drawn with size of 36 pixels and acreage of 2 mm^2^. Samples of ROIs in cortex, outer medulla, and inner medulla were shown in proper order from outer compartment to inner compartment of two kidneys (Fig. 3). Twelve pairs of ROIs were contoured to correspond to 6 sections for each rat. Accordingly, 24 pairs of ROIs were counted for each rat before and after injection, and there were 408 pairs of ROIs in total for all animals. The signal intensities of the renal cortex (SI_cortex_) and muscles (SI_muscle_) were measured and compared using an index of relative signal intensity (rSI): rSI = SI_cotex_/SI_muscle_. The change in rSI (ΔrSI) before and after injection was calculated using the following formula: ΔrSI = (rSI_post_—rSI_pre_)/rSI_pre_×100%, where rSI_post_ represents the rSI value measured after injection, and rSI_pre_ denotes the rSI value measured before injection [12,13]. Histograms of the kidney were performed with Functool software (GE Healthcare, ADW 4.4).

### Pathology

The animal model kidney specimens were cut into 1 mm^3^ cubes, fixed in a 2.5% glutaraldehyde solution, dehydrated using alcohol and embedded in an epoxy resin for sectioning. Ultra-thin slices were stained with uranyl acetate and lead citrate to observe the GBM and GEC foot processes of the kidneys with TEM. Prussian blue (PB) staining was performed as follows to observe the iron nanoparticle deposits. Sections were processed for PB staining and incubated for 30 min with 2% potassium ferrocyanide in 6% hydrochloric acid. The sections were counterstained with nuclear fast red and viewed under a light microscope. The cells with iron deposits were counted, measuring the iron nanoparticles deposited in the glomeruli under a visual field at 200x magnification using ordinary microscopy. For immunofluorescence of C5b-9 expression, sections of the kidneys were snap frozen in optimum cutting temperature (OCT) medium. The 4-μm thick tissue slices were cut, washed with PBS three times and stained with fluorescein isothiocyanate (FITC)-labeled rabbit anti-human anti-C5b-9 (Biosynthesis Biotechnology Co., Beijing, China). Tissue slices were assessed using a fluorescent microscope (TE2000; Nikon, Japan).

### Statistical Analysis

TheΔrSI values of the renal cortex for each group are presented as the means ± SD. The Spearson test and linear regression were used for correlation analysis between the iron nanoparticle counting and ΔrSI. One-way ANOVA was performed for comparisons of ΔrSI between groups. All data analyses were performed using SPSS 16.0 software (SPSS, Inc., Chicago, IL, USA). A *p* value of <0.05 was considered statistically significant.

## Results

### Probe characterization

**Fig 4:**
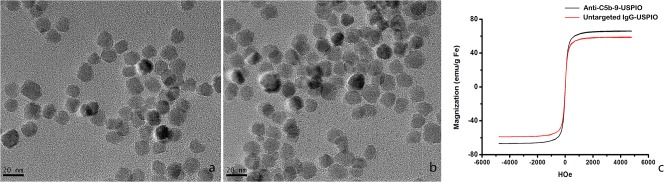
Characterization of the USPIOs. TEM image of the nanoparticles showed that the pure USPIO (a) and anti-C5b-9-USPIO (b) were fine and granular with an even size distribution. Fig. 4C showed the magnetization curve of anti-C5b-9-USPIO and IgG-USPIO nanoparticles at room temperature. The saturation magnetization values of anti-C5b-9-USPIO and IgG-USPIO were 50 emu/mg and 55 emu/mg, respectively.

Transmission electron microscopy (TEM) showed that the USPIO nanoparticles were fine granular particles with even size. The diameters of these particles were 13.1±1.3 nm (Fig. 4, A). The targeting anti-C5b-9-USPIO probe (Fig. 4, B) and the untargeted probe, IgG-USPIO, were brown in the PBS solution, and no obvious sediments were observed. Table 1 lists the physical and chemical characteristics of the three types of nanoparticles. At room temperature, the saturation magnetization values of the anti-C5b-9-USPIO and IgG-USPIO probes were 50 emu/mg and 55 emu/mg, respectively (Fig. 4, C). The concentration of antibody per USPIO was about 50 μg protein/mg Fe.

**Table 1:** Physical and chemical characteristics of nanoparticles.

Formulations	Anti-C5b-9-USPIO	IgG-USPIO	Pure USPIO
Size (hydrodynamic diameter, nm)	38.4 ± 0.4	38.4 ± 0.7	28.9 ± 0.6
R1 at 128 MHz (s mmol/L)	1.08 ± 0.11	1.31 ± 0.02	1.38 ± 0.07
R2 at 128 MHz (s mmol/L)	223.50 ± 5.27	238.84 ± 5.76	292.74 ± 5.91

All values are expressed as the mean ± SD. All sizes are based on the weighted averages. The relaxivities listed were obtained in PBS at 128 MHz and 25°C. R1 = longitudinal relaxation rate; R2 = transverse relaxation rate; USPIO = ultrasmall superparamagnetic iron oxide.

### Changes in biochemical parameters in urine and plasma of the rats

The biochemical parameters are shown in Table 2. The urinary protein excretion of the model group was 21.26±3.40 mg/24 h urine, which was significantly higher than the excretion of the normal group (*p*＜0.01). The serum albumin level in the model group showed a significant decrease compared to the normal group (*p*＜0.05). The serum creatinine, total cholesterol (TG) and blood urea nitrogen (BUN) remained in normal levels. The results above indicated that the pathological changes in kidneys of the model group were at an early stage, which were confirmed by transmission electron microscopy of the kidneys.

**Table 2:** Biochemical parameters in urine and plasma of the model group and nomal group.

Groups	urinary protein (mg/24 h urine)	serum albumin (g/L)	serum total cholesterol (mmol/L)	serum creatinine (μmol/L)	blood urea nitrogen (mmol/L)
**Model group**	21.69±3.40	40.79±2.56	1.38±0.21	70.32±10.35	7.91±0.91
**Control group**	3.91±1.98	47.01±3.12	1.31±0.15	72.66±11.26	8.08±0.80

### Pathological findings in animal models

**Fig 5:**
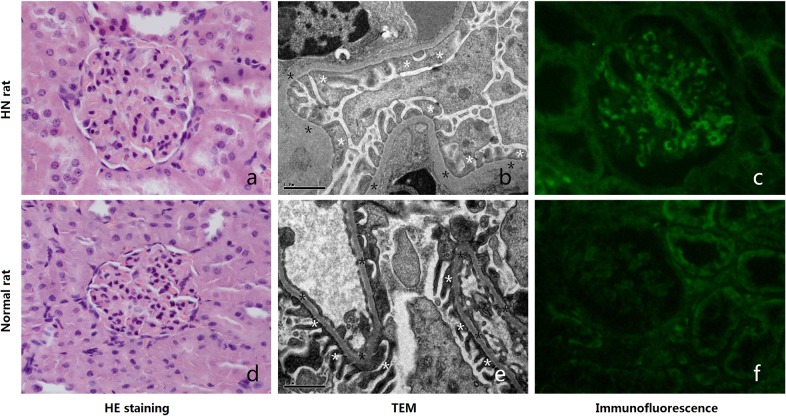
Pathology for HN model and normal rats. Histological images (HE staining, 400×) showed no obvious changes in glomerulus in HN rats (a) compared to normal rats (d). TEM (12500×) images of the kidneys showed segmental thickening of the GBM (b, black asterisk) and foot process fusion (b, white asterisk) in an HN rat. GBM (e, black asterisk) thickening and foot process (e, white asterisk) fusion were not observed in normal rats. Immunofluorescence showed C5b-9 depositions along the GBM in a glomerulus of HN rats (c), and no C5b-9 deposition in normal rats (f).

Histological findings with HE staining showed that no obvious changes were observed in glomerulus of HN rats (Fig. 5,A) compared to normal rats (Fig. 5,D). TEM showed segmental thickening of the glomerular basement membrane (GBM) (Fig. 5, B, black asterisk) and fusion of some GEC foot processes (Fig. 5, B, white asterisk) in the model group (Heymann nephritis) with pathological staging between HN Stage I and Stage II. The control group clearly demonstrated foot processes with no GBM thickening (Fig. 5, E, black asterisk) or fusion of GEC foot processes (Fig. 5, E, white asterisk). Immunofluorescence microscopy confirmed the abundant deposition of C5b-9 in the glomerulus and a few of tubules in HN rats (Fig. 5, C), and no C5b-9 depositions in normal rats (Fig. 5, F).

### *In vivo* MRI results

**Fig 6:**
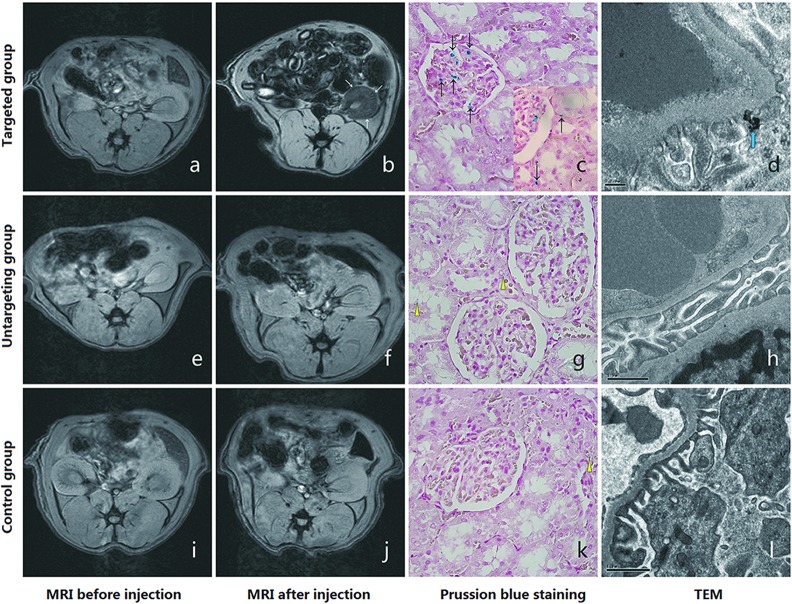
*In vivo* MRI and pathology of the three groups. T2*-weighted imaging was used in the three groups (n = 5, respectively). The signal intensities of the kidneys decreased significantly in the targeting group after injection (b, white arrow) compared to before injection (a). Diffusive iron particle depositions in the glomerulus (c, 400×, black arrow) and a few iron particles deposited in renal tubules (c, 400×, right corner) are demonstrated. TEM confirmed iron particles (d, 30000×, blue arrow) deposited under foot process. No significant signal intensity changes were observed in the kidneys of the untargeted and control groups between before and after injection. Iron particle depositions in the glomeruli of the untargeted group (g, 400×) and control group (k, 400×) were not observed. However, several iron particles were deposited in some renal tubules (g and k, yellow triangle) in both groups. TEM confirmed the absence of iron particle deposition under foot process (h and l, 12500×).

**Fig 7:**
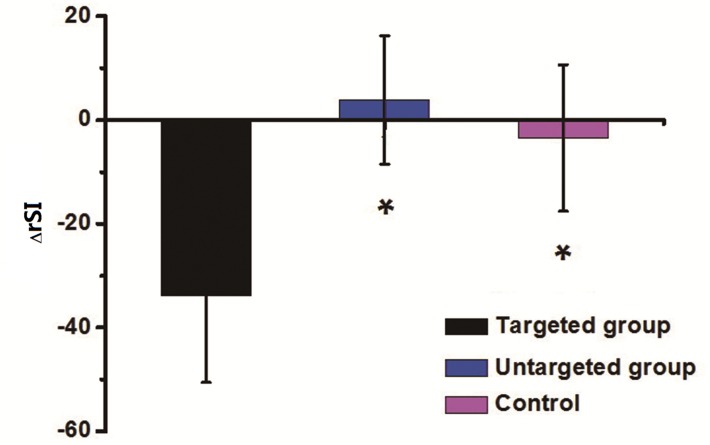
The changes in rSI (ΔrSI) of the three groups. The rSI decreased remarkably in targeting group compared to the other two groups. The differences in ΔrSI between the targeting group and untargeted group or control group were also statistically significant (*: *p*<0.05, v.s. targeted group).

Twenty-four hours after injecting the MRI probe, the signal intensities of kidneys in the targeting group decreased in all compartments (Fig. 6, B); the ΔrSI was approximately −34%±16.7%, −26%±13.9% and −16%±15.0% in the cortex, outer medulla and inner medulla, respectively. The signal intensities of kidneys in the untargeted (Fig. 6, E and F) and control (Fig. 6, I and J) groups exhibited no significant changes before and after injections, having ΔrSI values of 3.9±12.4% and −3.5±14.1%, respectively. The relative signal intensity (rSI) and the changes in rSI (ΔrSI) of the three groups were shown in Fig. 7. The differences in ΔrSI between the targeting group and untargeted group or control group were statistically significant (the *p* values were 0.001 and 0.006 respectively). The difference in ΔrSI between the untargeted group and the control group was not statistically significant (the *p* value was 0.441).

**Fig 8:**
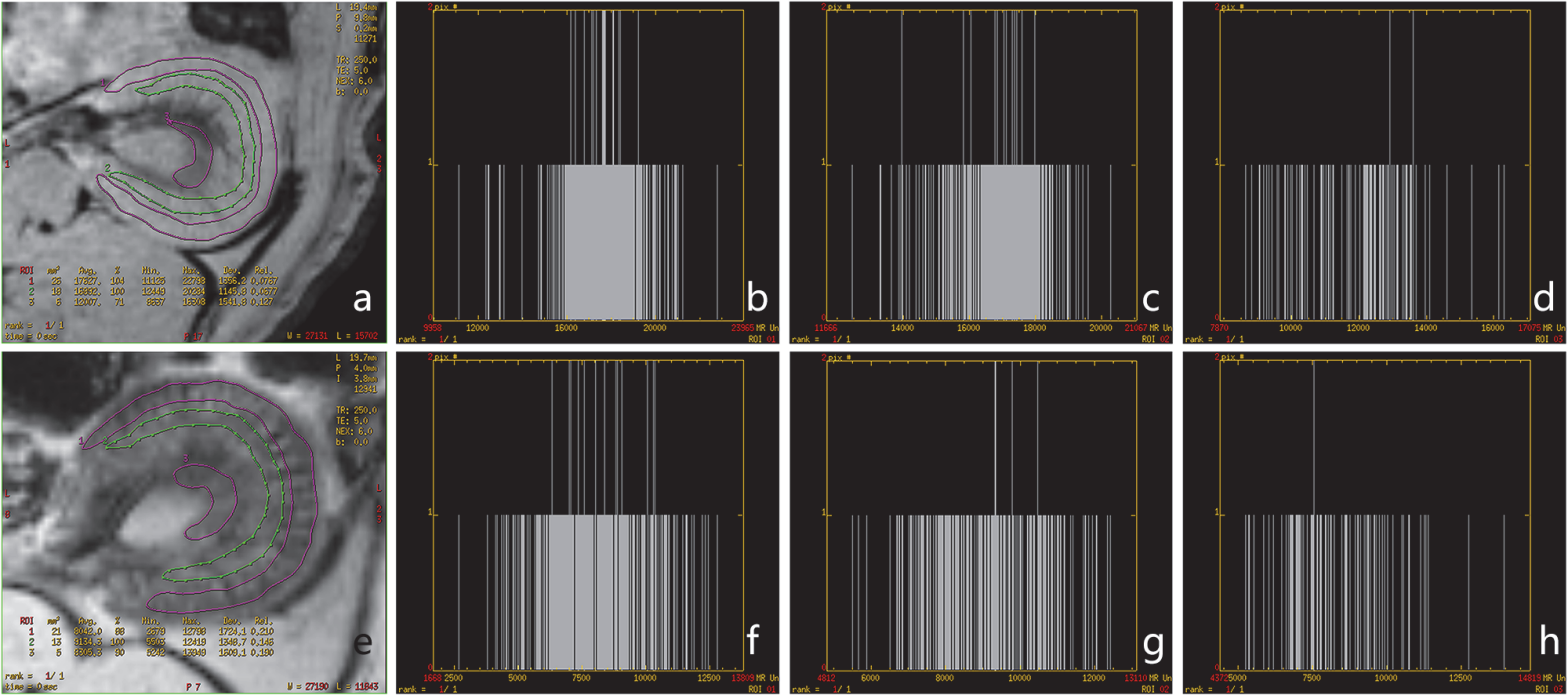
Histograms of the signal intensity distributions in one case of the targeting group. The average MR unit values of the cortex (ROI 1), outer medulla (ROI 2) and inner medulla (ROI 3) decreased significantly after probe injection. X-axis, MR unit values of pixels. Y-axis, counts of pixels.

The signal intensity distributions of one kidney in the targeting group were illustrated in Fig. 8. The histograms in Fig. 8 showed that the MR signal intensities in all parts of the kidney decreased significantly after probe injection. The mean MR unit value before injection was 17627, 16892, and 12007 in the cortex (ROI 1), outer medulla (ROI 2) and inner medulla (ROI 3), respectively. By contrast, the mean values of the three portions decreased significantly after injection, with the values of 8042.0, 9134.3, and 8305.3, respectively. The pixel count of each MR unit and the distribution ranges of the MR units were also displayed in Fig. 8. The range of MR unit value of the cortex before injection (ROI 1) was from 11125 to 22798 (Fig. 8B). In comparison, the range after injection was significantly lower than before injection (Fig. 8F) with values from 2679 to 12798. Similar changes were found in the outer medulla and inner medulla, as shown in Fig. 8C, 8G and Fig. 8D, 8H, respectively.

### Pathological findings after *In vivo* MRI Scan

The pathological findings of kidneys following Prussian blue staining showed diffuse iron particle depositions in the glomeruli (black arrow) of HN rats in the targeting group and few deposits in the renal tubules (Fig. 6, C). An example of iron particle deposition under foot processes is depicted in Fig. 6D using electron microscopy. Only several iron particles in some renal tubules (triangle) and no deposits in the glomeruli (Fig. 6, G and K) were observed in the untargeted and control groups. TEM confirmed the absence of iron particles deposition under foot process (Fig. 6, H and L).

## Discussion

Molecular imaging is a widely applied, useful tool for studying various tumors, tracking stem cells after transplantation and monitoring atherosclerotic plaques [9,14,15]. Molecular imaging studies in the kidneys have mainly focused on tracking transplanted stem cells in patients with kidney failure, and few studies have reported on nephropathy, including nephrotic syndrome and glomerulonephritis. Sargsyan et al. and Serkova et al. [16,17] introduced a complement-targeting complex labeled with ferric oxide nanoparticles in their investigations, and this nanoparticle complex-based imaging was feasible for evaluating pathological changes in the kidneys of mice with lupus.

Several studies have reported the use of USPIO for the evaluation of nephropathy. However, the observed deposits of iron nanoparticles in these reports were mostly due to macrophage phagocytosis, which was induced by the inflammatory response [18,19,20,21]. In contrast, the MN model applied in our investigation specifically represents glomerulonephritis with immune complex deposits under the foot processes or the GBM, and this model lacks the inflammatory response and inflammatory cell infiltration. To our knowledge, no studies have reported the use of USPIO to evaluate MN.

MN is the main pathological nephrotic syndrome in adults, and C5b-9-induced injury of cytomembranes is an important factor in MN pathogenesis. Complement-derived C5b-9 might be inserted into GEC membranes, which increases cell permeability resulting in an imbalance in the osmotic pressure between the inside and outside of cells. Consequently, intracellular signal transduction pathways are activated. Certain inflammatory mediators and cytokines are synthesized and released, which results in secondary injury, cellular apoptosis and glomerulosclerosis. Studies have confirmed the presence of C5b-9 depositions under GECs and inside GBMs [3,5]. HN is a classic MN disease model, and reports have shown that HN pathogenesis is similar to human MN. Moreover, the elucidations of MN pathogenesis and proteinuria primarily are concluded from study of HN [4,5]. Therefore, our study applied the HN model in rats.

We chose the C5b-9 complex, a key factor in MN, as an imaging target. A C5b-9-targeting MRI probe (anti-C5b-9-USPIO) was synthesized by combining a C5b-9 antibody and USPIO. The USPIO nanoparticles applied in this study were generated using “one-pot” reaction technology and modified by bio-soluble carboxyl PEG (α, ω-dicarboxyl-terminated PEG[HOOC-PEG-COOH], M.W. = 2000) with good hydrophilicity and a relatively long circulation time. Previous reports have confirmed the practical applications of the USPIO nanoparticles [22,23].

Our *in vivo* MRI results indicated that the signal intensities of the kidneys, and especially of the cortex, were reduced significantly after probe injection in the targeting group compared to the other two groups, consistent with pathological findings in the kidneys. The pathological basis of the significant signal decrease in kidney cortex is that glomerulus are mostly located in renal cortex, and glomerulus injury is the characteristic feature of Heymann nephritis. The reduced renal medulla signal in the targeting group was most likely the result of C5b-9 deposition in the tubular brush border. As previously reported [24,25], C5b-9 deposits mainly under the foot processes and partially in the tubular brush border. This pattern was confirmed in our experiments; iron particles deposits were greater in renal glomeruli than in tubules.

The present initial study suggests that anti-C5b-9-USPIO can be used as a novel C5b-9-targeting probe for molecular imaging in rats with HN. This approach can be applied for the early diagnosis of nephropathy because the pathological staging of HN models used in our study was between Stage I and Stage II. In addition, this probe can also evaluate the renal state using quantitative MRI. The major contribution of our work was to provide a practical and targeted molecular imaging method for the study of MN. Moreover, C5b-9 is also deposited in other types of nephritis, such as IgA nephropathy, anaphylatic purpura nephritis, membranous hyperplastic nephritis, et al. It has been proved that the detection of C5b-9 in urine and plasma is much more important to membranous nephropathy and IgA nephropathy. The molecular imaging method used in our experiment may be applied in other nephropathy relative with C5b-9 deposition.

The present study has some limitations. First, anti-C5b-9 is a biological macromolecule with a large molecular weight, complex structure and immunogenicity and may not be appropriate for use in humans. Recent reports [26,27] have shown that an affibody may be a potential substitute in future studies because affibodies are functionally similar to antibodies but display certain advantages, including a small molecular weight, weak immunogenicity, strong tissue penetration and high affinity. Due to the higher affinity towards specific molecular targets than peptides and smaller size compared to antibodies, affibody molecules are attractive candidates as a promising platform for targeting imaging and therapy applications [28,29,30]. The positive results in our study were preliminary; however, the feasibility of the targeting MR imaging in membranous nephropathy was demonstrated for the first time, to our knowledge, and this increased our confidence in further research using more biocompatible substances like affibodies. Synthesis of affibodies and exploration of their use in membranous nephropathy are next goals in our near future work. Second, the dynamic monitoring of nanoparticle deposits was not available in this study due to logistic difficulties in arranging scanning time, and only one scan was performed 24 hours after the injection. The dynamic monitoring of nanoparticle deposits and the potential injury to kidney are our experiment objective in the future.
